# Asprosin promotes vascular inflammation via TLR4-NFκB-mediated NLRP3 inflammasome activation in hypertension

**DOI:** 10.1016/j.heliyon.2024.e31659

**Published:** 2024-05-23

**Authors:** Rui Ge, Jun-Liu Chen, Fen Zheng, Shu-Min Yin, Min Dai, Yi-Ming Wang, Qi Chen, Yue-Hua Li, Guo-Qing Zhu, Ai-Dong Chen

**Affiliations:** a Key Laboratory of Targeted Intervention of Cardiovascular Disease, Collaborative Innovation Center for Cardiovascular Disease Translational Medicine, and Department of Physiology, Nanjing Medical University, Nanjing, Jiangsu, 211166, China; b Department of Pathophysiology, Nanjing Medical University, Nanjing, Jiangsu, 211166, China

**Keywords:** Asprosin, Inflammasome, Hypertension, Vascular remodeling

## Abstract

**Objective:**

and design Mild vascular inflammation promotes the pathogenesis of hypertension. Asprosin, a newly discovered adipokine, is closely associated with metabolic diseases. We hypothesized that asprosin might led to vascular inflammation in hypertension via NLRP3 inflammasome formation. This study shows the importance of asprosin in the vascular inflammation of hypertension.

**Methods:**

Primary vascular smooth muscle cells (VSMCs) were obtained from the aorta of animals, including spontaneously hypertensive rats (SHR), Wistar–Kyoto rats (WKY), NLRP3^−/−^ and wild-type mice. Studies were performed in VSMCs *in vitro*, as well as WKY and SHR *in vivo*.

**Results:**

Asprosin expressions were up-regulated in VSMCs and media of arteries in SHR. Asprosin overexpression promoted NLRP3 inflammasome activation via Toll-like receptor 4 (TLR4), accompanied with activation of NFκB signaling pathway in VSMCs. Exogenous asprosin protein showed similar roles in promoting NLRP3 inflammasome activation. Knockdown of asprosin restrained NLRP3 inflammasome and p65-NFκB activation in VSMCs of SHR. NLRP3 inhibitor MCC950 or NFκB inhibitor BAY11-7082 attenuated asprosin-caused VSMC proliferation and migration. Asprosin-induced interleukin-1β production, proliferation and migration were attenuated in NLRP3^−/−^ VSMCs. Local asprosin knockdown in common carotid artery of SHR attenuated inflammation and vascular remodeling.

**Conclusions:**

Asprosin promoted NLRP3 inflammasome activation in VSMCs by TLR4-NFκB pathway, and thereby stimulates VSMCs proliferation, migration, and vascular remodeling of SHR.

## Highlights

• Asprosin promotes NLRP3 inflammasome activation, which contributes to the VSMC proliferation and migration.
• The effects of asprosin on the VSMCs are mediated by activating TLR4/NF-κB signaling pathway.
• Inhibition of asprosin expression in arteries attenuates vascular inflammation and vascular remodeling in hypertension.

## Graphical abstract

**Figure:**
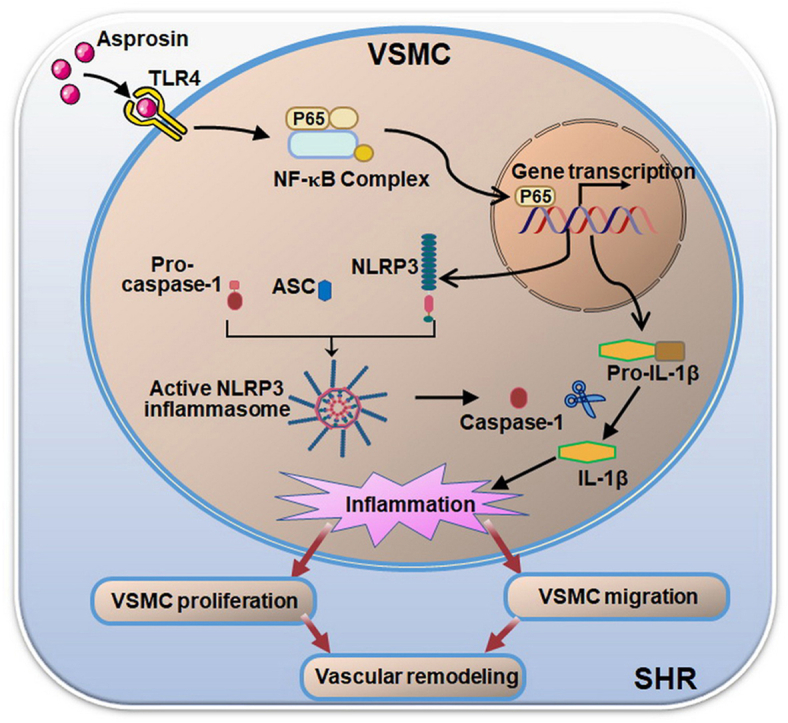

## Abbreviations

FBN1: fibrillin-1
IL-1β: interleukin-1β
KD: knockdown
MA: mesenteric artery
NC: negative control
NFκB: nuclear factor κB
NLRP3: NOD-like receptor thermal protein domain associated protein 3
OE: overexpression
PFU: plaque forming units
SHR: spontaneously hypertensive rats
TLR4: toll-like receptor 4
VSMCs: vascular smooth muscle cells
WT: wild type
WKY: Wistar–Kyoto rats

## 1 Introduction

Vascular remodeling progressively aggravates hypertension and organ damage [[1], [2], [3]]. Proliferation and migration of vascular smooth muscle cells (VSMCs) are crucial for vascular remodeling [4,5]. Chronic vascular inflammation contributes to hypertension [6,7]. Innate and adaptive immunity promotes hypertension by causing vascular inflammation and remodeling, and chronically mild inflammation in hypertension is marked by the increased inflammatory mediators in arteries [8]. NOD-like receptor thermal protein domain associated protein 3 (NLRP3) inflammasome is responsible for the production of proinflammatory factors in response to external stimuli [9]. The inflammasome is composed of NLRP3, ASC and caspase-1. The inflammasome assembling promotes the conversion of pro-interleukin (IL)-1β to IL-1β, and then causes inflammation [10]. Typically, the activation of NLRP3 requires activation signals involving toll-like receptors (TLRs) to activate NFκB [11]. The inflammasome stimulates VSMCs phenotypic transition and vascular remodeling in hypertension [12]. NLRP3 knockout mitigates vascular remodeling in hypertension [7]. NLRP3 inflammasome might be a feasible therapeutic target for hypertension [13].

Asprosin is an adipokine closely correlated with metabolism regulation and metabolic diseases [[14], [15], [16], [17]]. Asprosin widely distributes in various tissues and cells [18,19], and functions as paracrine or circulating hormone [20]. Asprosin proprotein can be cleaved into fibrillin-1 (FBN1) and asprosin [21]. Asprosin triggers hepatic glucose release by cAMP-PKA signaling [22], and is upregulated in humans and animals with obesity [23]. Inhibition of asprosin production attenuates hyperinsulinism in the mice with diet-induced obesity [[24], [25], [26]]. We have shown that asprosin in hypothalamic paraventricular nucleus increases sympathetic activity and blood pressure [27]. It has been found that asprosin impairs insulin secretion, and serves as a pro-inflammatory player in macrophages via TLR4 pathway [28]. Asprosin promotes hyperlipidemia-induced endothelium inflammation through NFκB signaling [29]. However, the roles of asprosin in vascular inflammation are almost unknown. We hypothesized that asprosin could promote inflammation in VSMCs and arteries and contribute to vascular remodeling in hypertension. The aims of this study were to determine the roles of asprosin in vascular inflammation remodeling in hypertension.

## 2 Methods

### 2.1 Rats and mice

Male Wistar-Kyoto rats (WKY) and spontaneously hypertensive rats (SHR) were purchased from Vital River Laboratory Animal Technology Co. Ltd (Beijing, China). NLRP3^−/−^ and wild-type (WT) mice were obtained from The Jackson Laboratory (Bar Harbor, ME, USA). Animals were euthanized with pentobarbital sodium (200 mg/kg, i.v.).

### 2.2 Rat and mouse VSMC culture

Rat and mouse primary VSMCs were prepared from thoracic aorta of 8-week-old WKY and SHR, as well as 12-week old WT and NLRP3^−/−^ mice as we previously described [7,30]. The 2nd to 5th generations of VSMCs were used in the study. For the experiment of dose-effects of asprosin, 95 % confluent VSMCs were respectively exposed to various asprosin concentrations (0. 12.5, 25, 50 and 100 nM) for 48 h.

### 2.3 Overexpression and knockdown in VSMCs

Overexpression (OE) and knockdown (KD) were performed as we previously reported [31]. Plasmids and small interfering RNA (siRNA) were commercially obtained from RiboBio (Guangzhou, Guangdong, China). The siRNA sequence was shown in a supplementary table (Table S1).

### 2.4 Local asprosin knockdown in carotid artery

Adenovirus transfection with siRNA for asprosin KD was performed in rat carotid arteries. Recombinant adenoviruses for asprosin KD and negative control adenoviruses were obtained from Genechem (Shanghai, China). The siRNA sequence was shown in Table S1. Pluronic F127 (PF127) is a non-ionic surfactant that enhances cell permeability, and is relatively non-toxic to cells without altering the membrane properties of cells. It is soluble at low temperature, but forms a gel at room temperature. PF127 is a gel for the control of chemical diffusion to surrounding tissues without cell toxicity [32]. PF127 at a concentration of 30 % has been used in the local carotid artery region for promoting chemical delivery and limiting the chemical diffusion to other tissues after forming a gel [[33], [34], [35]]. Surgery was performed under anesthesia (pentobarbital sodium, 50 mg/kg, i.p.) and aseptic technique. Unilateral carotid artery was exposed and separated from adjacent tissues by a piece of Parafilm (Heathrow Scientific, Vernon Hills, IL, USA). 30 % PF127 (Sigma, St Louis, MO, USA) solution containing PBS, Ad-NC or Ad-asprosin (2 × 10^8^ PFU/mL) was dripped on the surface of the carotid artery 3 times (15 μL for each administration) with an interval of 10 min. Parafilm was removed 30 min after the last administration of the gel, and the wound was closed. Tail-cuff system was used for measuring blood pressure every week [36].

### 2.5 Immunofluorescence staining

For VSMCs, the cells were incubated with anti-NLRP3 (1:100) antibody, anti-ASC (1:100) or anti-p65 antibody (1:100). For arteries, the sections were incubated with anti-asprosin (1:100) or α-SMA antibody (1:200). CY3-conjugated goat anti-rabbit IgG (1:200) or FITC-conjugated monkey anti-goat IgG (1:100) was used as second antibody. DAPI was used for nuclear staining. Fluorescence microscopy (Axiolab5, Zeiss, Jena, Germany) or confocal laser scanning microscope (Lsm880, Zeiss) was used to detect the fluorescence.

#### 2.6 VSMC proliferation and migration assays

VSMC proliferation and migration were evaluated by EdU incorporation assay and Boyden chamber assay, respectively, as we previously reported [37,38].

### 2.7 HE staining and Masson's staining

Carotid arteries were fixed, sectioned, and prepared for HE staining or Masson's staining with standard protocols. The images were obtained with a light microscope (BX-51, Olympus, Tokyo, Japan). Vascular remodeling was evaluated with the media thickness, lumen diameter and the ratio of media thickness to lumen diameter.

### 2.8 qRT-PCR

Total RNAs were extracted with Trizol reagent (Vazyme, Nanjing, Jiangsu, China). Reverse transcription was performed with a PrimeScript RT reagent Kit (Vazyme) using 1 μg total RNA with a PrimeScript RT reagent Kit. qRT-PCR was done with SYBR Green Master Mix (Vazyme) on StepOnePlus™ Real-Time PCR System (Applied Biosystems, Foster City, CA, USA). GAPDH was employed as an internal control. Primers are shown in supplementary table (Table 2).

### 2.9 Western blotting

Western blot analysis was performed with standard protocols. The dilution of antibodies for the measurements are listed as follows. NLRP3 antibody (No. DF15549), 1:1000; ASC antibody (No. 10500-1-AP), 1:1000; caspase-1 antibody (No. 81482-1-RR), 1:1000; IL-1β antibody (No. ab283818), 1:1000; toll-like receptor 4 (TLR4) antibody (No. 30400-1-AP), 1:1000; p65 antibody (No. 10745-1-AP), 1:1000; HRP-conjugated second antibody (No. SA00001-2), 1:10000. Lamin B1 antibody (No. 12987-1-AP, 1:5000) or β-actin antibody (No. 20536-1-AP, 1:10000) was used as normalized control.

### 2.10 Chemicals and antibodies

Asprosin, MCC950 and BAY11-7082 were purchased from MedChem Express (Monmouth Junction, NJ, USA). Antibodies against p65, ASC, caspase-1, TLR4, lamin B1 and β-actin were purchased from Proteintech Group Inc (Chicago, IL, USA). NLRP3 antibodies were purchased from Affinity (Changzhou, Jiangsu, China). IL-1β antibody was obtained from Abcam (Cambridge, MA, USA). CY3-conjugated goat anti-rabbit IgG and FITC-conjugated monkey anti-goat IgG were obtained from Servicebio (Wuhan, Hubei, China).

### 2.11 Statistics

Experiments conformed to randomized and double-blinded principles. Two-way ANOVA and subsequent Bonferroni test were used for multiple comparisons. Student's unpaired *t*-test was used for comparison between two groups. All data are represented as mean ± SE. *P < 0.05* was considered significant.

## 3 Results

### 3.1 Asprosin expressions in arteries and VSMCs

**Fig. 1:**
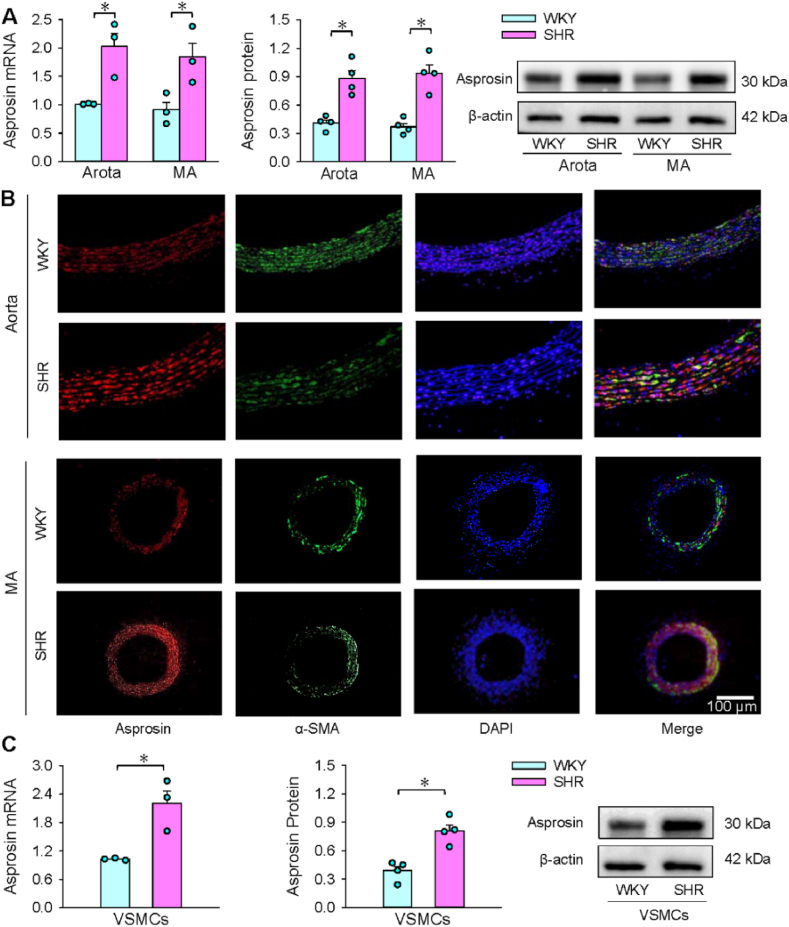
Asprosin expressions. A, asprosin mRNA and protein in aorta and MA. B, immunofluorescence staining in aorta and MA. Red, asprosin; Green, α-SMA; Blue, DAPI. C, asprosin mRNA and protein in VSMCs. Two-way ANOVA followed by Bonferroni test for A; Unpaired student's test for C. n = 3 or 4. *P < 0.05. (For interpretation of the references to colour in this figure legend, the reader is referred to the Web version of this article.)

Asprosin was upregulated in aorta and mesenteric artery of SHR (Fig. 1A). Immunofluorescence analysis showed that asprosin immunoreactivity in media of arteries were enhanced in SHR (Fig. 1B). Similarly, asprosin was also upregulated in VSMCs of SHR (Fig. 1C).

#### 3.2 Effects of asprosin OE and KD on VSMC inflammation

**Fig. 2:**
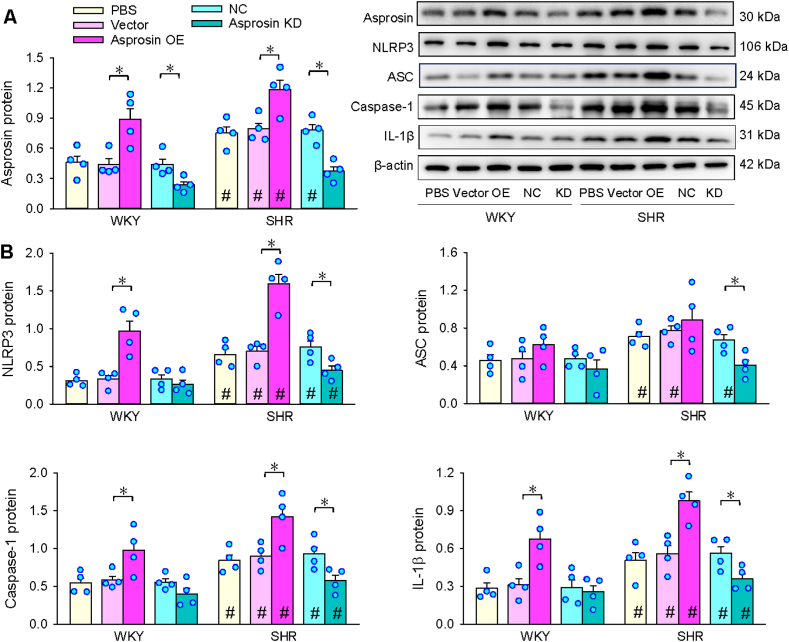
Effects of asprosin overexpression (OE) or knockdown (KD) on related protein expressions in VSMCs. A, asprosin protein. B, NLRP3, ASC, caspase-1, IL-1β protein expressions. Cells were incubated for 48 h with asprosin OE plasmid (2 μg) or asprosin siRNA (50 nM). Two-way ANOVA followed by Bonferroni test. n = 4. *P < 0.05; #P < 0.05 vs WKY.

Effectiveness of asprosin OE or KD was identified by corresponding changes of asprosin expression (Fig. 2A). NLRP3, ASC, caspase-1 and IL-1β in VSMCs were upregulated in SHR. Asprosin OE promoted NLRP3, caspase-1 and IL-1β expressions, but not ASC expression in WKY and SHR. Asprosin KD inhibited NLRP3, ASC, caspase-1 and IL-1β expressions in SHR, but not in WKY (Fig. 2B). The findings indicate that increased asprosin expression in VSMCs is responsible for NLRP3 inflammasome activation and inflammation in SHR.

### 3.3 Roles of asprosin protein in VSMC inflammation

**Fig. 3:**
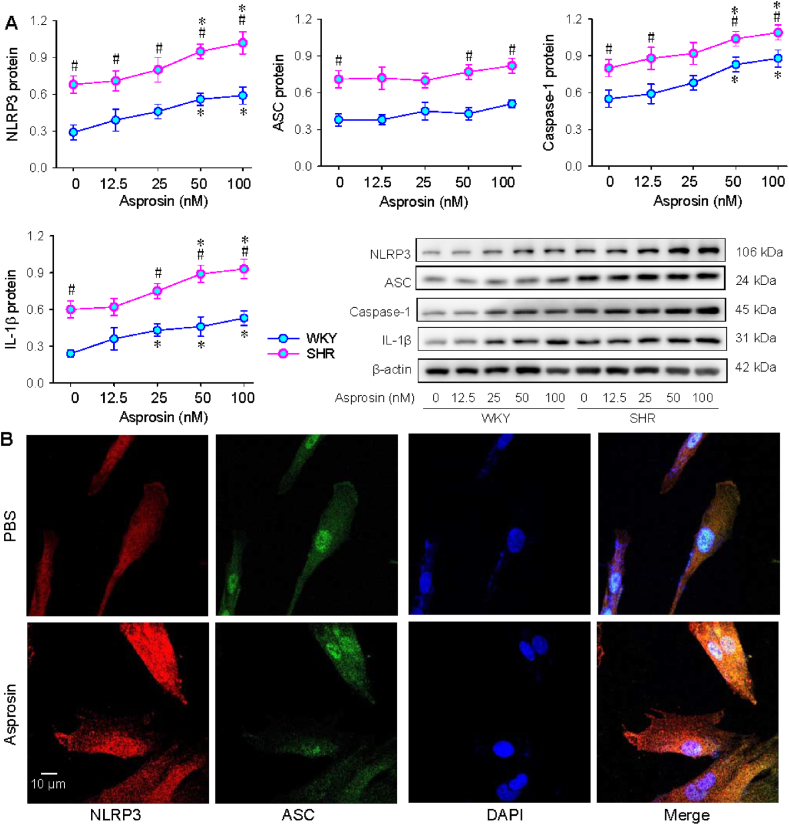
Effects of asprosin protein on NLRP3 inflammasome activation in VSMCs. A, dose-effects of asprosin protein (0. 12.5, 25, 50 and 100 nM) on NLRP3-related protein expressions in WKY and SHR. B, immunofluorescence showing the roles of asprosin (50 nM) on NLRP3 (red) and ASC (green) expressions in SHR. Two-way ANOVA followed by Bonferroni test. n = 4. *P < 0.05 vs 0 nM; #P < 0.05 vs WKY. (For interpretation of the references to colour in this figure legend, the reader is referred to the Web version of this article.)

Asprosin protein dose-relatedly increased NLRP3, caspase-1 and IL-1β expression but not ASC expression in VSMCs of WKY and SHR (Fig. 3A), which were similar to the effects of asprosin overexpression mentioned above. Asprosin promoted VSMC proliferation, almost reaching maximal effects at 50 nM level (Fig. S1). Immunofluorescence analysis confirmed that asprosin protein promotes NLRP3 expressions in SHR (Fig. 3B). The findings indicate that exogenous asprosin protein has similar roles to endogenous asprosin in VSMCs in promoting inflammasome activation and inflammation.

### 3.4 Asprosin receptors in VSMC inflammation

**Fig. 4:**
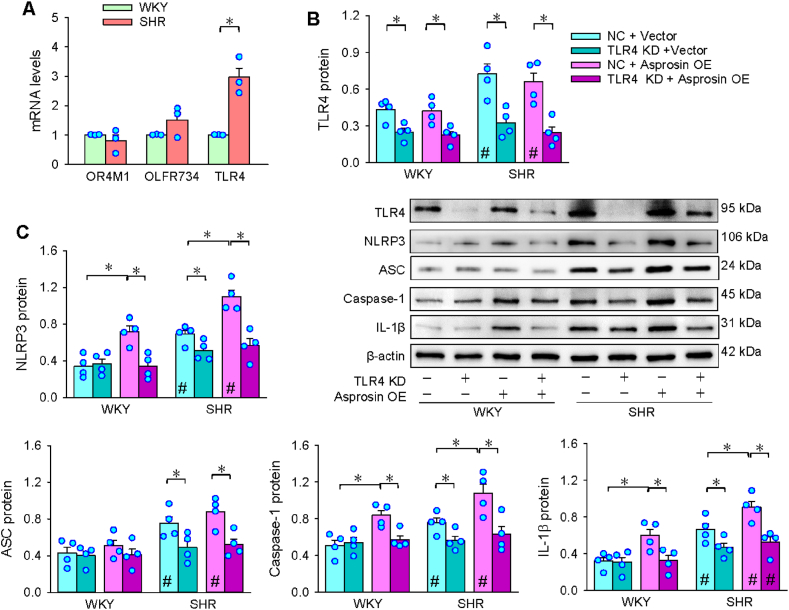
Asprosin receptors in VSMCs. A, mRNA levels of OR4M1, OLFR734 and TLR4. B and C, effects of asprosin receptor TLR4 KD on asprosin OE-induced NLRP3-related protein expressions. Cells were pretreated with TLR4 siRNA (50 nM) for 24 h before asprosin OE plasmid (2 μg) treatment for 24 h. Two-way ANOVA followed by Bonferroni test. n = 3 or 4. *P < 0.05; #P < 0.05 vs WKY.

OR4M1, OLFR734 and TLR4 may serve as asprosin receptors in regulating metabolism [22,39,40]. TLR4 mRNA level was upregulated in SHR, but not OR4M1 and OLFR734 mRNA levels (Fig. 4A), suggesting a possibility that the effects of asprosin on inflammation may be mediated by TLR4. The hypothesis is supported by previous findings that TLR4 is closely related to inflammation [[41], [42], [43]]. TLR4 KD significantly reduced the TLR4 expression, confirming the effectiveness of TLR4 KD. However, asprosin OE did not affect TLR4 protein expression, indicating that the TLR4 upregulation in SHR is independent on asprosin upregulation (Fig. 4B). TLR4 KD not only reduced the NLRP3, ASC, caspase-1 and IL-1β protein levels in SHR, but inhibited asprosin OE-caused upregulation of these proteins in WKY and SHR (Fig. 4C). The findings suggest that TLR4 mediates the effects of asprosin on NLRP3 inflammasome activation.

### 3.5 NFκB signaling in asprosin-induced VSMC inflammation

**Fig. 5:**
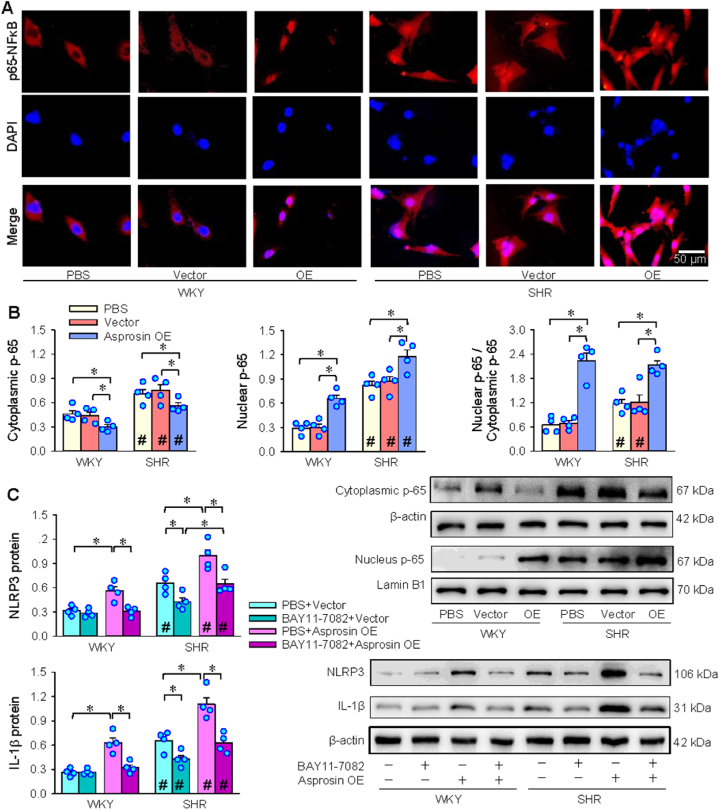
Role of NFκB in asprosin overexpression-induced NLRP3 inflammasome activation in VSMCs. A, immunofluorescence staining showing p65-NFκB nuclear translocation. Red, p65-NFκB; Blue, DAPI. B, intracellular and intranuclear p65-NFκB levels. C. Effects of a NFκB inhibitor BAY11-7082 (10 μM) on NLRP3 and IL-1β protein expressions. Two-way ANOVA followed by Bonferroni test. n = 4 or 6. *P < 0.05; #P < 0.05 vs WKY. (For interpretation of the references to colour in this figure legend, the reader is referred to the Web version of this article.)

Nuclear factor-κB (NFκB) signaling contributes to TLR4-mediated inflammation [[41], [42], [43]], and is involved in NLRP3 inflammasome activation in SHR [12]. It is interesting to know whether NFκB has an effect of asprosin on inflammation in VSMCs. In this study, immunofluorescence analysis showed that asprosin OE promoted p65-NFκB nucleus translocation in VSMCs (Fig. 5A). The asprosin overexpression-induced p65-NFκB nucleus translocation was confirmed by Western blotting (Fig. 5B). More importantly, NFκB inhibitor BAY11-7082 not only reduced NLRP3 and IL-1β expressions in SHR, but inhibited asprosin OE-induced NLRP3 and IL-1β upregulation in WKY and SHR (Fig. 5C). The findings suggest that p65-NFκB nucleus translocation contributes to the effects of asprosin on NLRP3 inflammasome activation and inflammation.

### 3.6 Roles of NLRP3 in the effects of asprosin on proliferation and migration

**Fig. 6:**
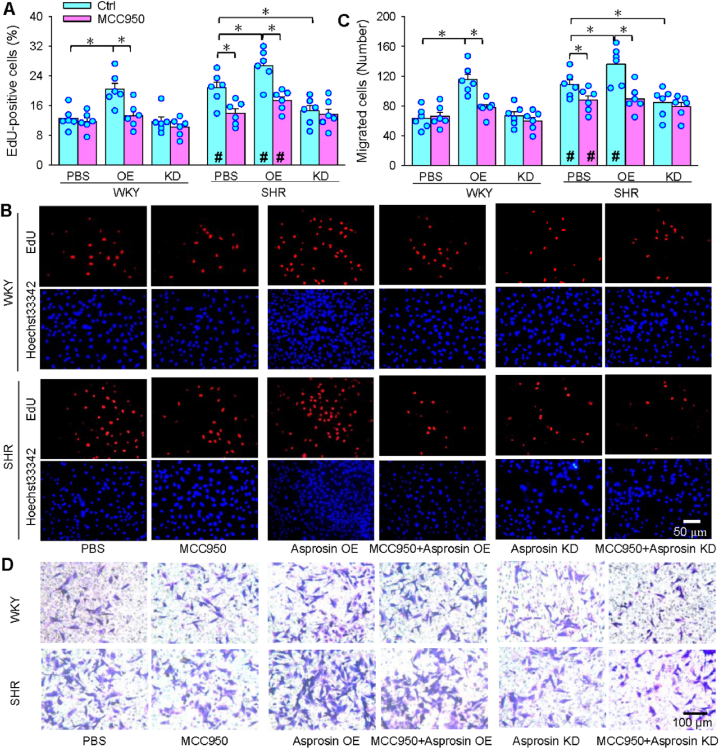
Effects of NLRP3 inflammasome inhibitor MCC950 (50 μM for 24 h) on asprosin OE-caused proliferation and migration of VSMCs. A-B, VSMC proliferation evaluated with EdU-positive cells (red). C-D, VSMC migration evaluated by Boyden chamber assay. Two-way ANOVA followed by Bonferroni test. n = 6. *P < 0.05; #P < 0.05 vs WKY. (For interpretation of the references to colour in this figure legend, the reader is referred to the Web version of this article.)

MCC950 is a NLRP3 inflammasome activation inhibitor [44]. MCC950 attenuated VSMC proliferation in SHR, and inhibited the effects of asprosin on VSMC proliferation in WKY and SHR (Fig. 6A and B). Similarly, MCC950 attenuated VSMC migration of SHR, and inhibited the effects of asprosin on migration in WKY and SHR (Fig. 6C and D). The findings suggest that NLRP3 inflammasome activation at least partially mediates the effects of asprosin.

### 3.7 Effects of NLRP3 deletion

**Fig. 7:**
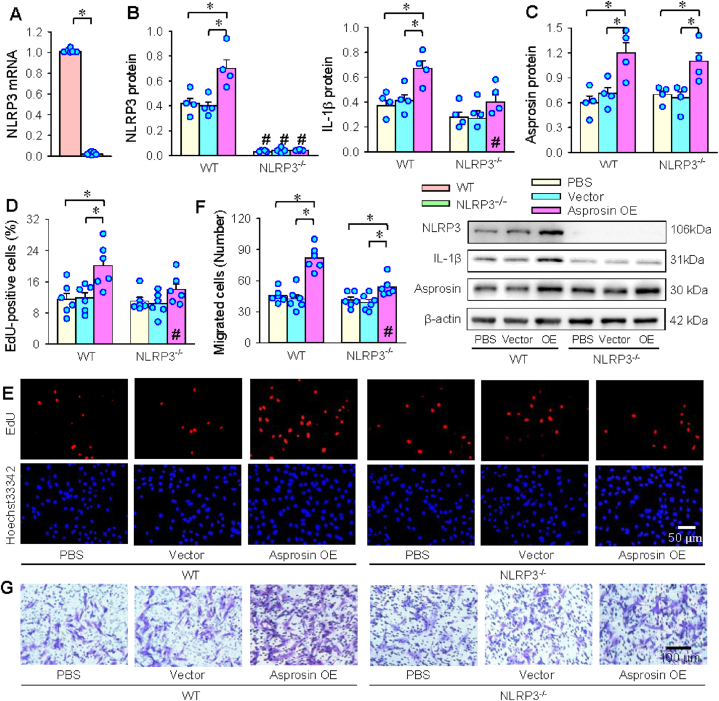
Effects of asprosin overexpression in VSMCs from WT and NLRP3^−/−^ mice. A, NLRP3 mRNA levels. B, NLRP3 and IL-1β protein expressions. C, asprosin protein expression. D-E, VSMC proliferation evaluated with the percentage of EdU-positive cells (red). F-G, VSMC migration evaluated by Boyden chamber assay. Values are mean ± SE. Unpaired student's test for A; Two-way ANOVA followed by Bonferroni test for B–F. n = 6 (A, D, E); n = 4 (B, C). *P < 0.05; #P < 0.05 vs WT. (For interpretation of the references to colour in this figure legend, the reader is referred to the Web version of this article.)

There were almost no NLRP3 expressions in the VSMCs of NLRP3^−/−^ mice (Fig. 7A and B). Asprosin OE increased NLRP3 and IL-1β expression in VSMCs of WT mice rather than those of NLRP3^−/−^ mice (Fig. 7B). Asprosin OE treatment effectively increased asprosin protein levels in both WT and NLRP3^−/−^ mice (Fig. 7C). However, asprosin OE promoted VSMC proliferation in WT mice, but not in NLRP3^−/−^ mice (Fig. 7D and E). Similarly, asprosin OE-induced VSMC migration was much weaker in NLRP3^−/−^ mice than WT mice (Fig. 7F and G). The findings indicate that NLRP3 inflammasome is crucial for the roles of asprosin in inducing VSMC inflammation, proliferation and migration.

### 3.8 Local asprosin KD in carotid artery on inflammation and vascular remodeling

**Fig. 8:**
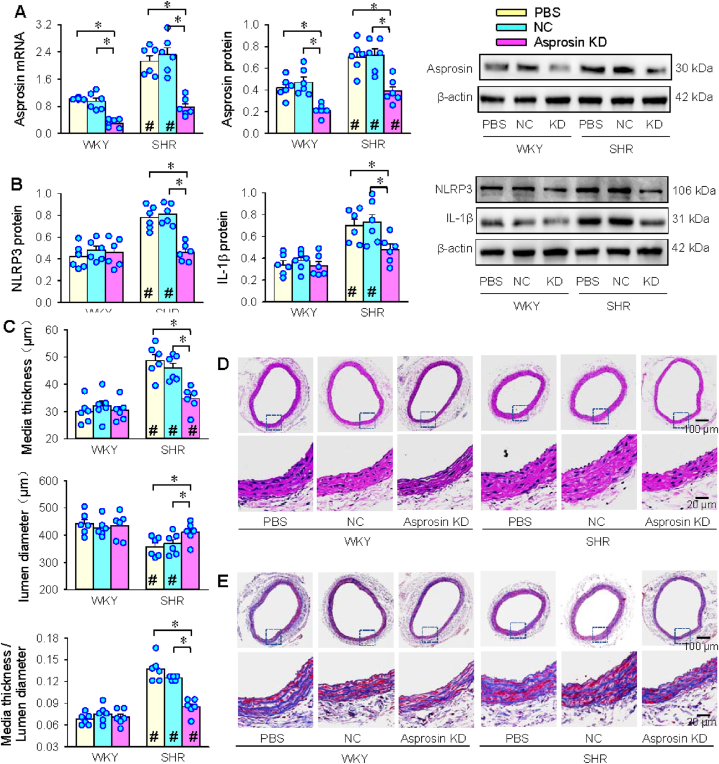
Local asprosin knockdown in common carotid artery attenuates vascular inflammation and remodeling in SHR. A, asprosin expressions in the carotid artery. B, NLRP3 and IL-1β expressions in carotid artery. C, bar graph showing the media thickness, lumen diameter and their ratio in carotid artery. D, hematoxylin-eosin (HE) staining of carotid artery. E, Masson's staining of carotid artery. Two-way ANOVA followed by Bonferroni test. n = 6. *P < 0.05; #P < 0.05 vs WKY.

Adenoviruses delivery of siRNA targeting asprosin was carried out in the local common carotid artery. Measurements were made 3 weeks after the transfection. Reduced asprosin expressions in carotid artery confirmed the effectiveness of local asprosin KD (Fig. 8A). Asprosin KD inhibited the NLRP3 and IL-1β expressions in VSMCs of SHR, but not in WKY (Fig. 8B). Both HE staining and Masson's staining of carotid artery showed that local asprosin KD increased lumen diameter, but reduced media thickness and media thickness/lumen diameter ratio in SHR (Fig. 8C–E). The findings suggest that asprosin is crucial for vascular inflammation and remodeling in SHR. The local asprosin KD in common carotid artery did not affect systolic blood pressure and heart rate (Fig. S2), suggesting that the effects of local asprosin knockdown were not secondary to the hemodynamic changes.

## 4 Discussion

Chronic and low-grade vascular inflammation is crucial for vascular remodeling in hypertension [6]. Asprosin is closely related to metabolic diseases [[14], [15], [16]]. However, the role of asprosin in vascular inflammation is not well known. Main new findings in the study are that asprosin promotes inflammation in VSMCs through TLR4/NFκB/NLRP3 pathway, which further contributes to vascular remodeling in hypertension. Suppression of asprosin in arteries alleviates vascular inflammation and remodeling.

NLRP3 inflammasome is involved in VSMC proliferation and vascular remodeling in hypertension [12]. It induces secretion of pro-inflammatory cytokines [45]. Circulating IL-1β level is increased in hypertensive patients [46]. We found that asprosin levels were increased in VSMCs, arteries of hypertensive rats compared with normotensive rats. Knockdown of asprosin attenuated NLRP3 inflammasome activation of SHR, while asprosin overexpression or exogenous asprosin protein promoted NLRP3 inflammasome activation in VSMCs of WKY and SHR. NLRP3 deletion abolished asprosin overexpression-caused IL-1β production in NLRP3^−/−^ mice. Chronic *in vivo* studies showed that adenovirus-mediated local asprosin knockdown in common carotid artery of hypertensive rats reduced the arterial NLRP3 expression and IL-1β production. These findings provide a solid evidence that asprosin serves as an important pro-inflammatory cytokine causing vascular inflammation in SHR. Increased asprosin expression is at least partially responsible for the enhanced chronic vascular inflammation in hypertension. The pro-inflammatory role of asprosin in VSMCs is supported by previous findings that asprosin promotes inflammation in macrophages and endothelium [29,47].

Previous studies suggest that three possible receptors OR4M1, OLFR734 and TLR4 may be involved in the roles of asprosin in metabolism regulation [22,39,40]. TLR4 pathway activation contributes to hypertension via inducing oxidative stress [48], and VSMCs proliferation of SHR by triggering the NLRP3 inflammasome [49]. We found that TLR4 mediated the effects of asprosin on NLRP3 inflammasome activation, which was supported by the findings that TLR4 is closely related to the inflammation [[41], [42], [43]]. It is interesting that asprosin overexpression had no effects on TLR4 expression, suggesting that TLR4 upregulation in SHR is not associated with the asprosin upregulation. It is known that NFκB mediates TLR4 activation-related inflammation [[41], [42], [43]]. We found that TLR4/NFκB pathway mediated asprosin-caused NLRP3 inflammasome activation and subsequent inflammation in VSMCs, which is supported by the findings that asprosin is involved in inflammation response and cellular dysfunction through binding to TLR4 receptors on mouse islet cells [50].

NLRP3 inflammasome activation promoted VSMC proliferation and vascular remodeling in mice with hypertension [7]. Either NLRP3 inflammasome inhibitor MCC950 or NLRP3 deletion prevented asprosin-caused VSMC proliferation, migration and inflammation. Chronic *in vivo* studies showed that adenovirus-mediated local asprosin knockdown in common carotid artery prevented the arterial remodeling in SHR. Upregulated vascular asprosin contributed to the augmented VSMC proliferation, migration and vascular remodeling in hypertension.

There is a major limitation in investigating the effects of systemic intervention on vascular remodeling, because it is hard to known whether the changes of vascular remodeling are caused by its direct effects or secondary effects. We proposed that systemic knockdown of asprosin might cause extensive effects in metabolism and cardiovascular activity, which might have a secondary effect on vascular inflammation and remodeling. To determine the direct roles of asprosin, local asprosin knockdown in common carotid artery was performed *in vivo* instead of systemic asprosin knockdown to avoid its secondary effects. We found that local knockdown of asprosin attenuated vascular inflammation and remodeling in SHR, which may be attributed to its direct roles in attenuating NLRP3 inflammasome activation and subsequent inflammation. Since asprosin was highly expressed in arteries and VSMCs of SHR, the effects of local asprosin knockdown may primarily act on the local artery. There are two limitations. (1) The effects of systemic asprosin knockdown on metabolism and other cardiovascular activity were not investigated; (2) FBN1 is the precursor of asprosin, and a structural component of microfibers that provides mechanical support. FBN1 dysregulation is involved in several diseases including cancers, renal and cardiovascular diseases [51]. Whether FBN1 is involved in vascular inflammation and inflammasome activation in hypertension needs further investigation.

In conclusion, asprosin promotes NLRP3 inflammasome activation and subsequent inflammation in VSMCs via TLR4/NFκB signaling. Vascular asprosin is increased in SHR, which is partially responsible for NLRP3 inflammasome activation and inflammation, followed by enhanced VSMC proliferation and migration, as well as vascular remodeling. Asprosin plays crucial roles in vascular inflammation and remodeling. Asprosin is a potential therapeutically target for vascular inflammation and vascular remodeling in hypertension.

## Data availability statement

Data will be provided by the corresponding author upon request.

## Ethics declarations

The study was approved by Experimental Animal Care and Use Committee of Nanjing Medical University (IACUC-2306034).

## CRediT authorship contribution statement

**Rui Ge:** Writing – original draft, Investigation, Formal analysis. **Jun-Liu Chen:** Investigation, Formal analysis. **Fen Zheng:** Investigation, Formal analysis. **Shu-Min Yin:** Formal analysis, Investigation. **Min Dai:** Formal analysis, Investigation. **Yi-Ming Wang:** Investigation, Formal analysis. **Qi Chen:** Writing – review & editing, Conceptualization. **Yue-Hua Li:** Writing – review & editing, Conceptualization. **Guo-Qing Zhu:** Writing – review & editing, Writing – original draft, Visualization, Conceptualization, Formal analysis, Supervision, Validation. **Ai-Dong Chen:** Writing – review & editing, Writing – original draft, Conceptualization, Formal analysis, Supervision, Validation, Visualization.

## Declaration of competing interest

The authors declare that they have no known competing financial interests or personal relationships that could have appeared to influence the work reported in this paper.
