# Supplementary material for: Asprosin promotes vascular inflammation via TLR4-NFκB-mediated NLRP3 inflammasome activation in hypertension
Source: Heliyon. 2024 May 23;10(11):e31659. doi: 10.1016/j.heliyon.2024.e31659 (PMC11152944; doi:10.1016/j.heliyon.2024.e31659)
Supplement: Multimedia component 1 [file mmc1.pdf]

Online supplementary materials

(2 figures and 2 tables)

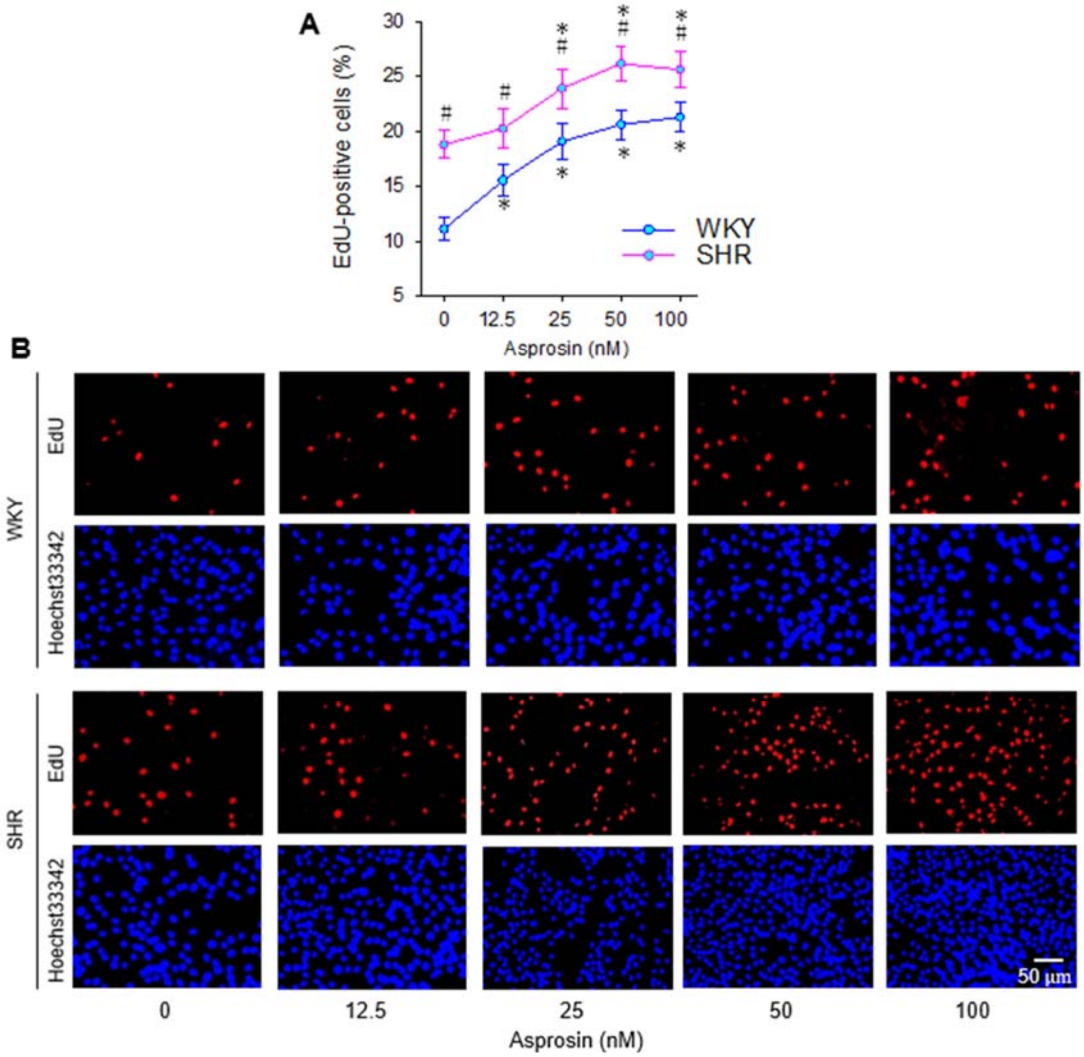

Figure S1 Effects of asprosin (0, 12.5, 25, 50 and 100 nM) on VSMCs proliferation of WKY and SHR. The proliferation was evaluated by the percentage of EdU-positive cells. A, Line graph showing the percentage of EdU-positive cells. B, Representative images showing the EdU-positive cells (red) and nuclei (blue) in the VSMCs of WKY and SHR. Values are mean±SE. * $P<0.05$ vs 0 nM; # $P<0.05$ vs. WKY. n=6. Two-way ANOVA followed by a Bonferroni post hoc test.

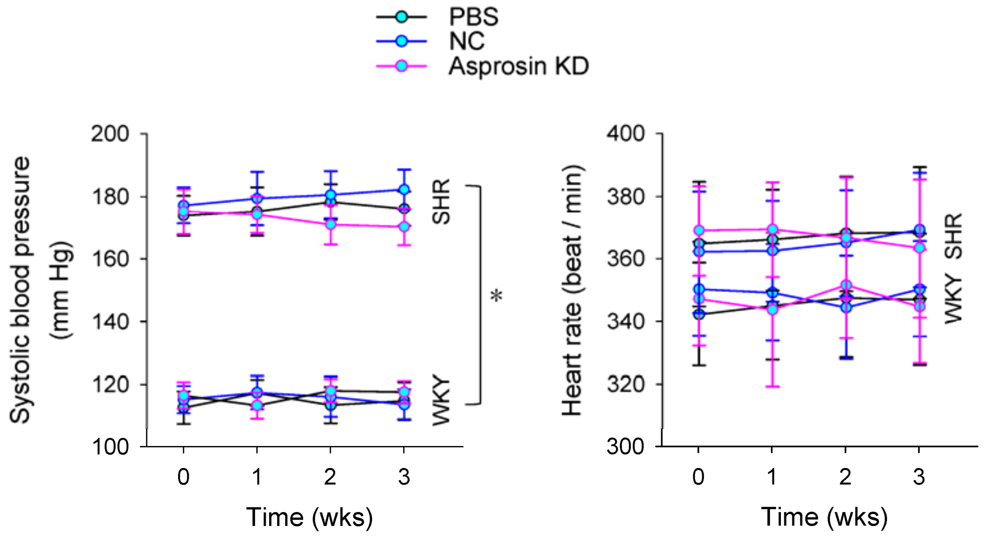

Figure S2 Effects of local asprosin KD in common carotid artery on systolic pressure and heart rate in WKY and SHR. Values are mean±SE. * P<0.05. n=6, Two-way ANOVA followed by a Bonferroni post hoc test.

Table S1 Sequences of siRNAs targeting rat asprosin and TLR4 mRNAs

siRNAs	siRNA sequences		Target sequences
Asprosin	Sense	5'- CAACUCUGAUGAACCACAA -3'	CAACTCUGATGAACCACAA
	Antisense	5'- UUGUGGUUCAUCAGAGUUG -3'	
TLR4	Sense	5'- CCUACCAAGUCUCAGCUAU -3'	CCTACCAAGTCTCAGCTAT
	Antisense	5'- AUAGCUGAGACUUGGUAGG -3'	

Table S2 Primers for RT-PCR analysis

Name	Primer	Sequence
Rat Asprosin	Forward	5’- TGCATGGTACAATCATTCACCG -3’
	Reverse	5’- CCAACTTCCGCCGATACTCA -3’
Rat TLR4	Forward	5’- CCGCTCTGGCATCATCTTCA -3’
	Reverse	5’- CTCCCACTCGAGGTAGGTGT -3’
Rat OLFR734	Forward	5’- CTCGGGAAGTGCAGCTAGTC -3’
	Reverse	5’- GCGGTCAAAAGCCATCACTG -3’
Rat GAPDH	Forward	5’- GAAGCTGGTCATCAACGGGA -3’
	Reverse	5’- ACGACATACTCAGCACCAGC -3’
Rat OR4M1	Forward	5’- GCAAATGATACCACCGTGACC -3’
	Reverse	5’- AAGACTAGCTGCACTTCCCG -3’
Mouse NLRP3	Forward	5’- CCTTAAGCTGGAGCTGCTGT -3’
	Reverse	5’- TCACCTCTCGGCAGTGGATA -3’
Mouse GADPH	Forward	5’- CAGGAGAGTGTTTCCTCGTCC -3’
	Reverse	5’- GAGGTCAATGAAGGGGTCGTT -3’
