# Supplementary material for: Asprosin promotes vascular inflammation via TLR4-NFκB-mediated NLRP3 inflammasome activation in hypertension
Source: Heliyon. 2024 May 23;10(11):e31659. doi: 10.1016/j.heliyon.2024.e31659 (PMC11152944; doi:10.1016/j.heliyon.2024.e31659)
Supplement: Multimedia component 2 [file mmc2.pdf]

Fig 1A

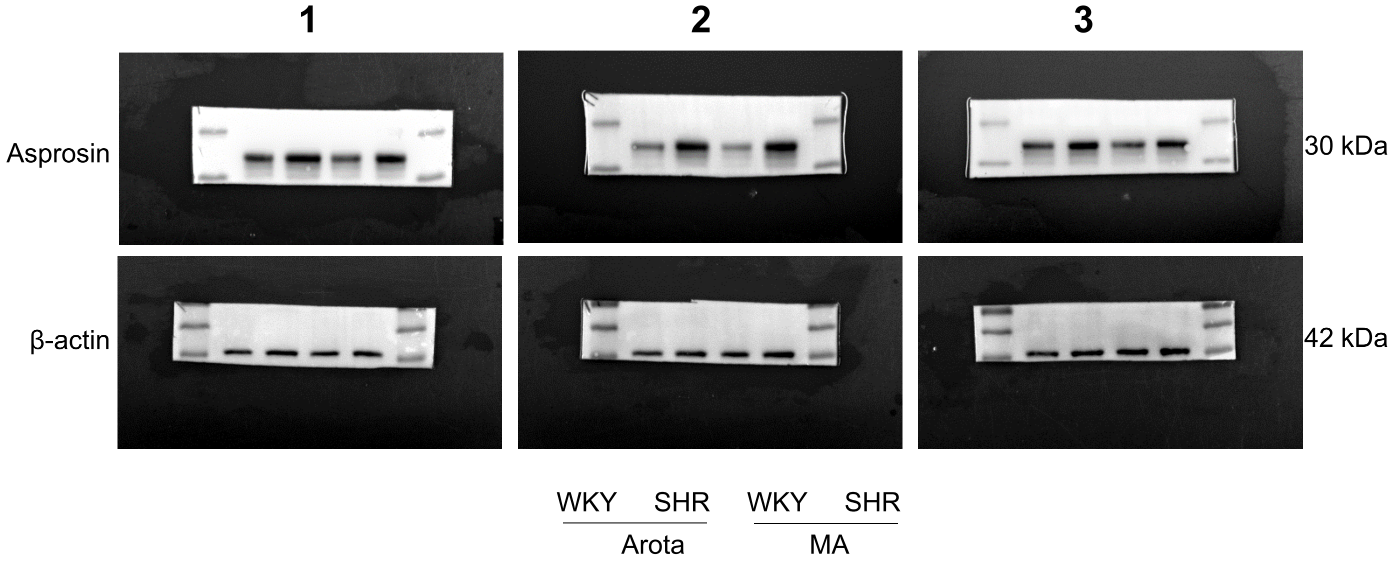
1
2
3
Asprosin
β-actin
30 kDa
42 kDa
WKY SHR WKY SHR
Arota
MA

Fig 1C

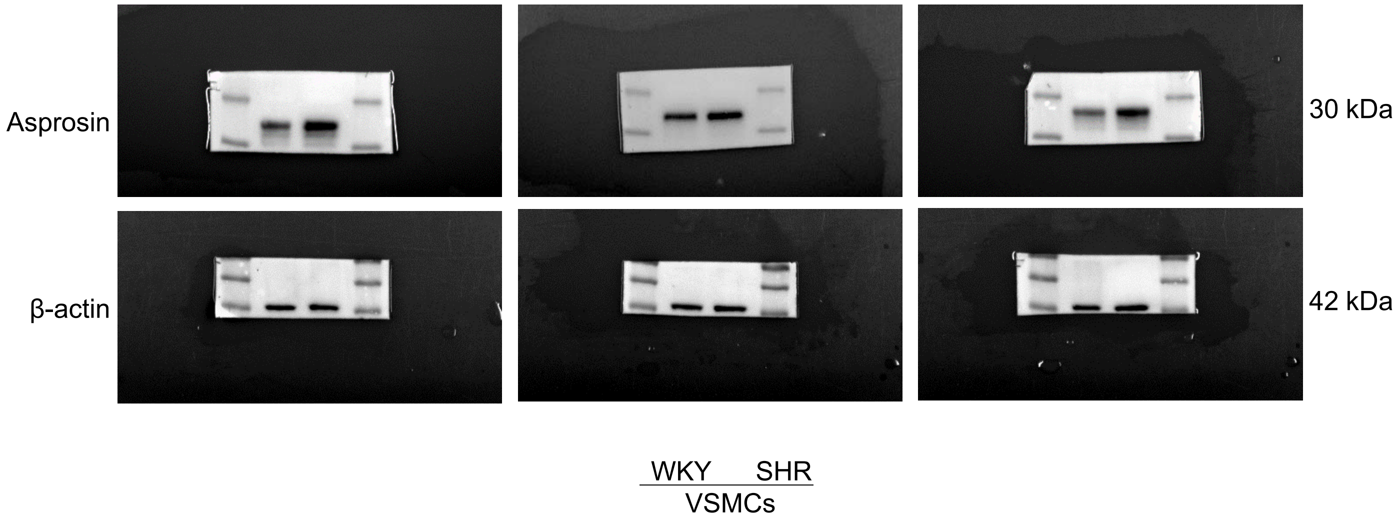
Asprosin
β-actin
30 kDa
42 kDa
WKY SHR
VSMCs

Fig 2A

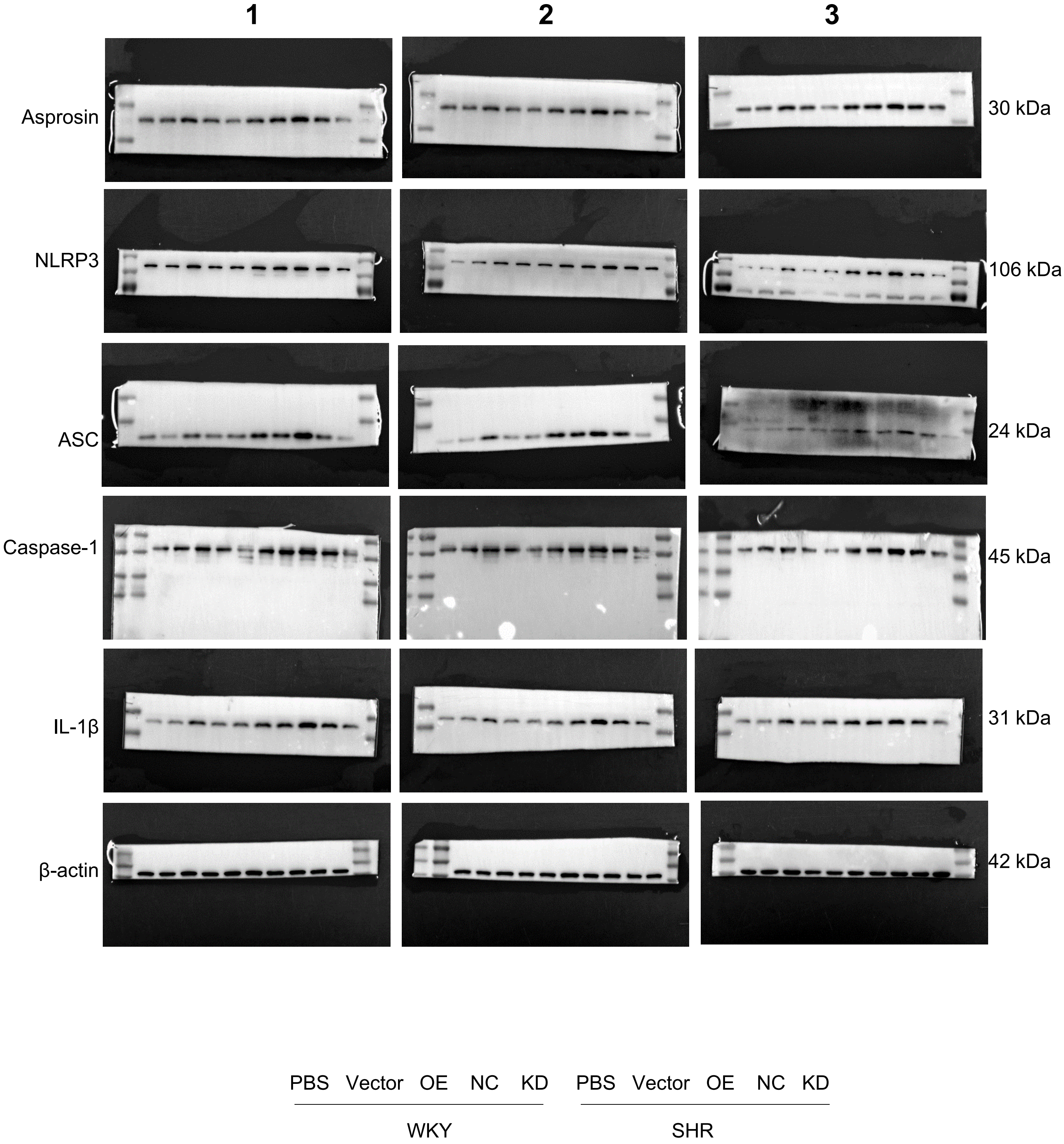
1
2
3
Asprosin
NLRP3
ASC
Caspase-1
IL-1β
β-actin
30 kDa
106 kDa
24 kDa
45 kDa
31 kDa
42 kDa
PBS Vector OE NC KD
PBS Vector OE NC KD
WKY
SHR

Fig 3A

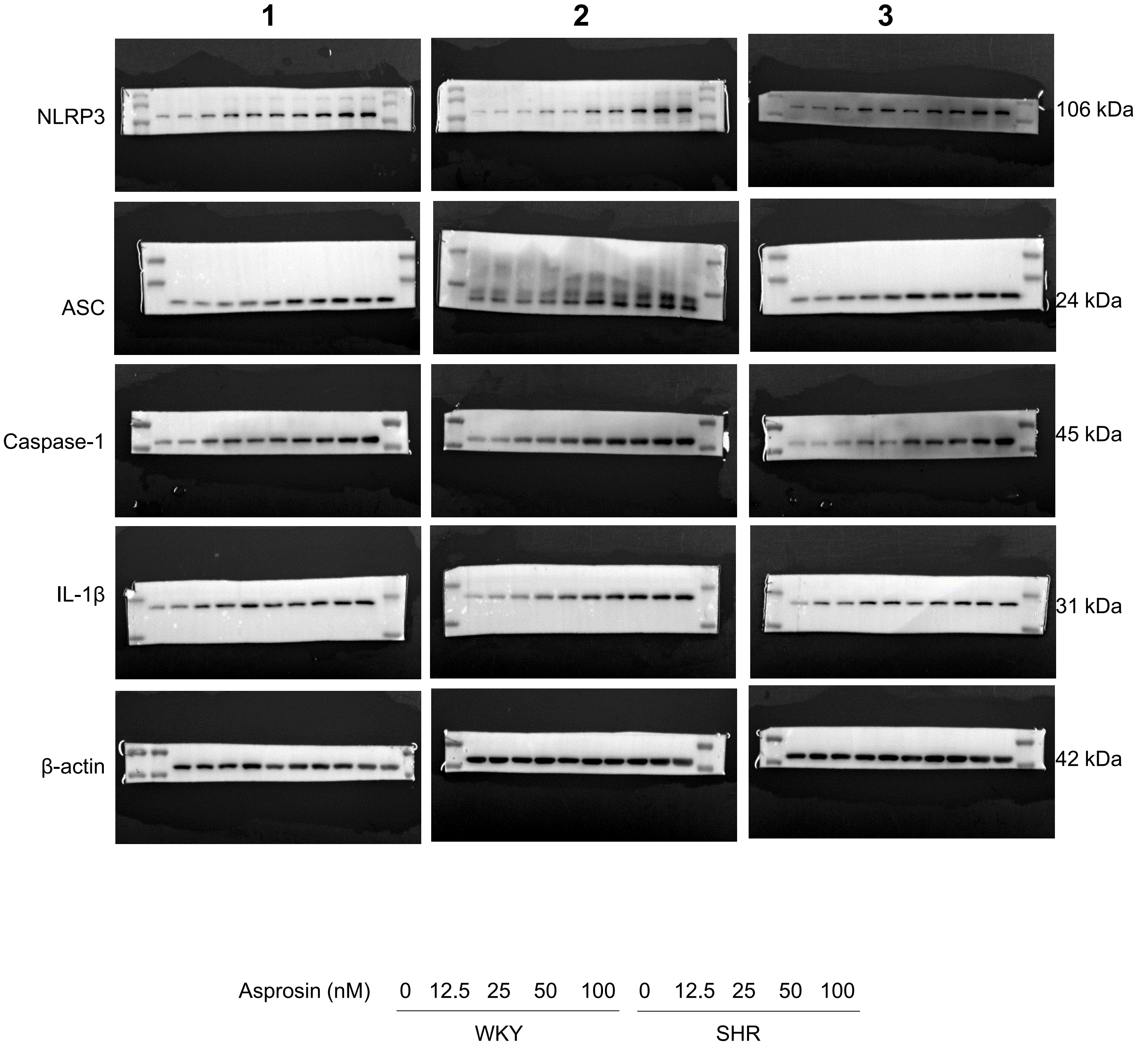

1
2
3
NLRP3
ASC
Caspase-1
IL-1β
β-actin
106 kDa
24 kDa
45 kDa
31 kDa
42 kDa
Asprosin (nM) 0 12.5 25 50 100 0 12.5 25 50 100
WKY
SHR

Fig 4B & C

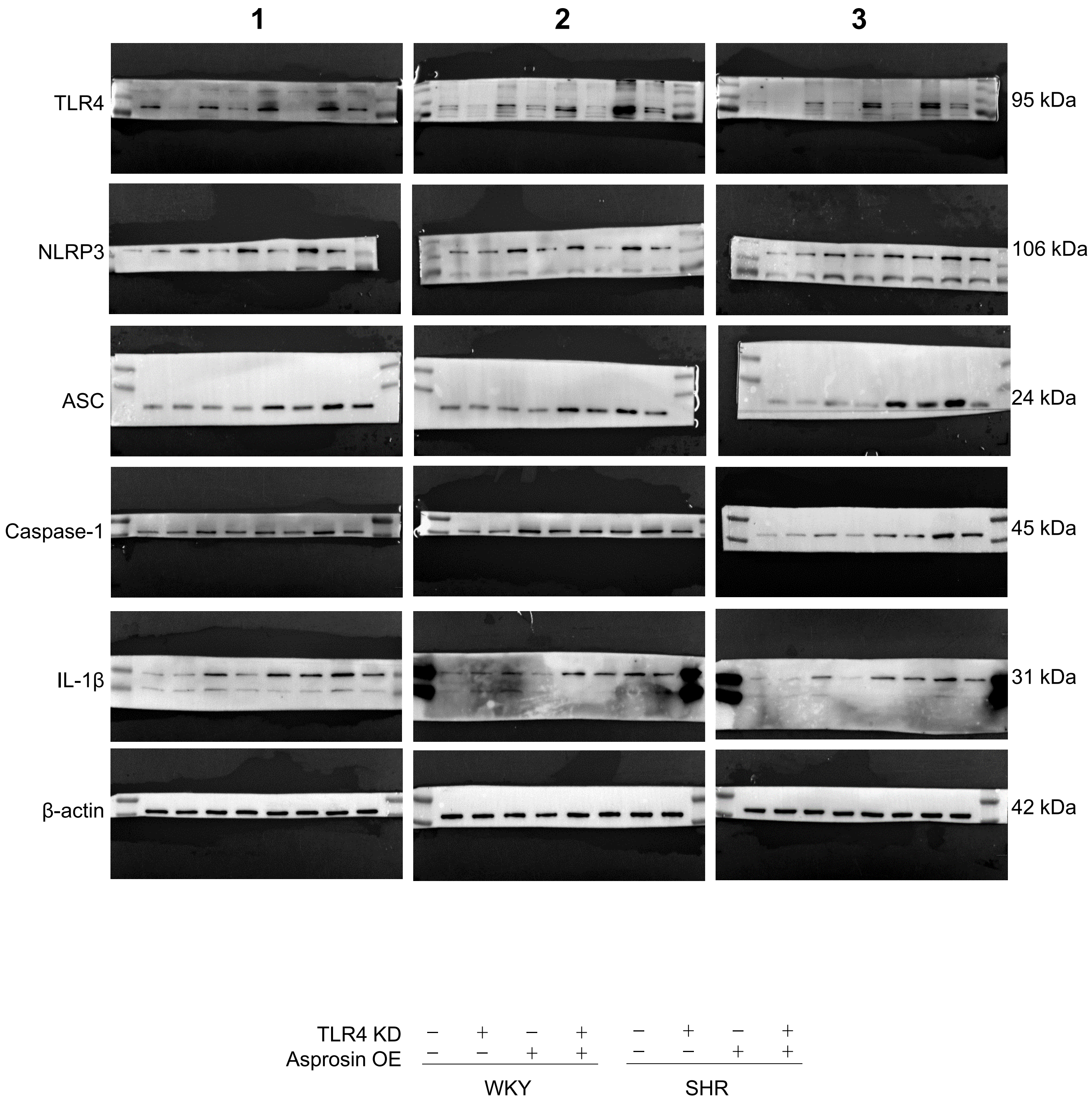

1
2
3
TLR4
NLRP3
ASC
Caspase-1
IL-1β
β-actin
95 kDa
106 kDa
24 kDa
45 kDa
31 kDa
42 kDa
TLR4 KD − + − + − + − +
Asprosin OE − − + + − − + +
WKY
SHR

Fig 5B

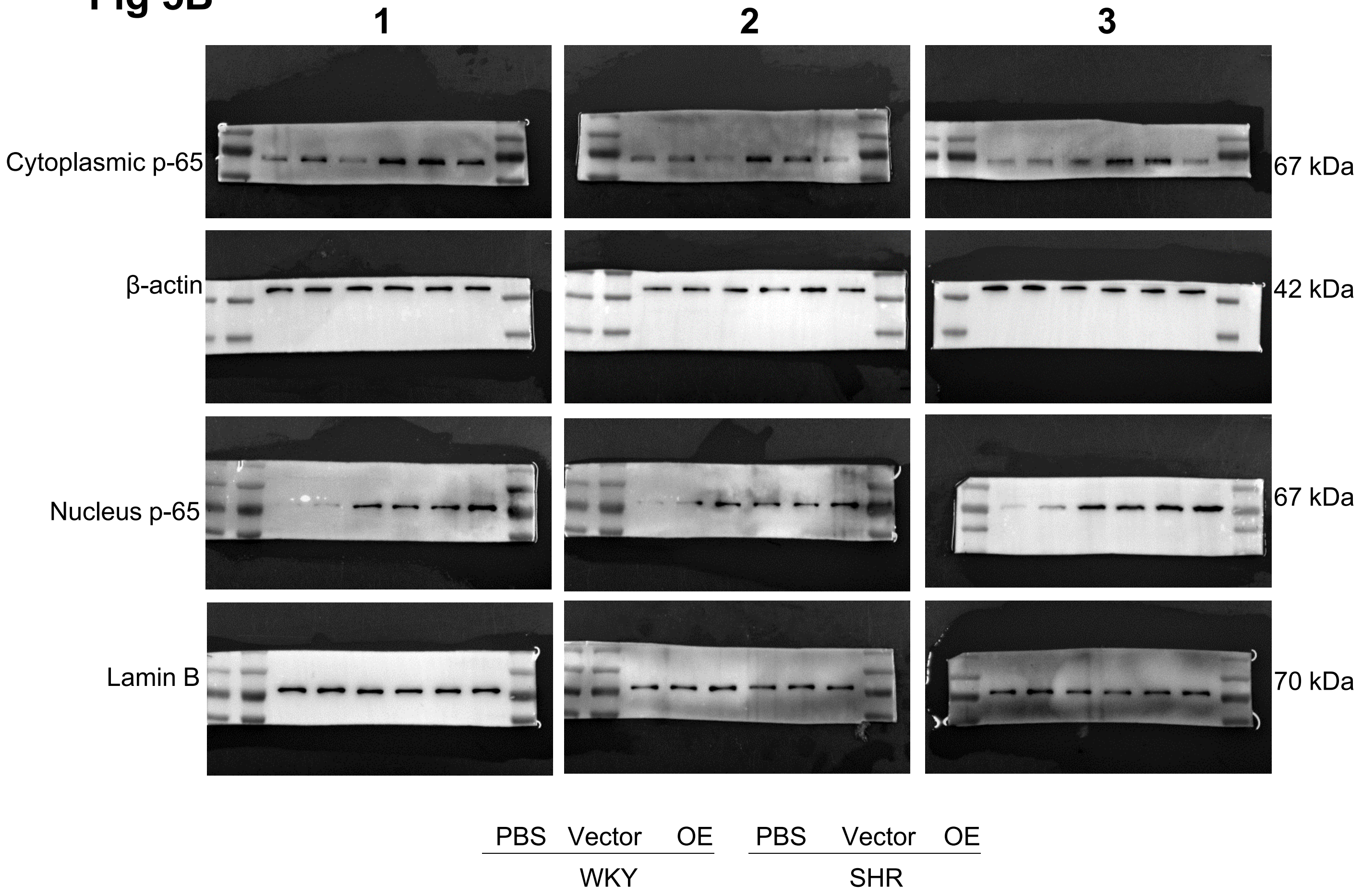
1
2
3
Cytoplasmic p-65
67 kDa
β-actin
42 kDa
Nucleus p-65
67 kDa
Lamin B
70 kDa
PBS Vector OE
WKY
PBS Vector OE
SHR

Fig 5C

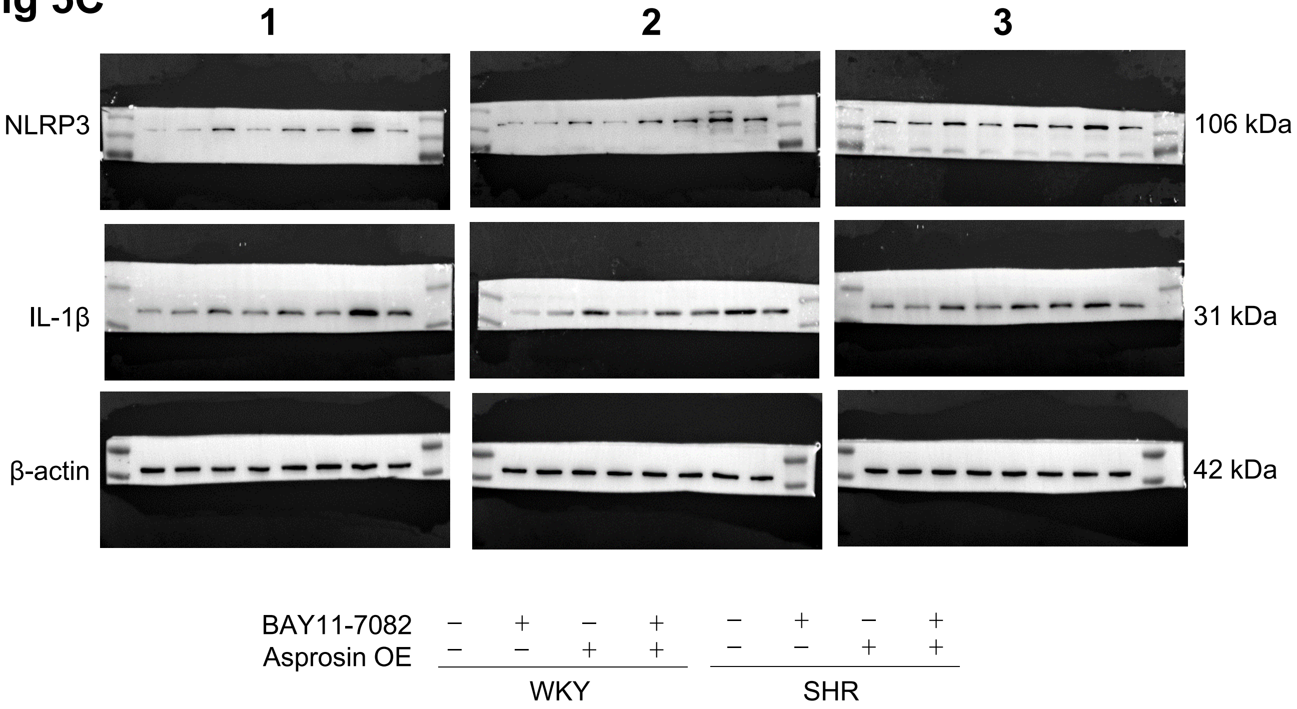
1
2
3
NLRP3
106 kDa
IL-1β
31 kDa
β-actin
42 kDa
BAY11-7082 − + − + − + − +
Asprosin OE − − + + − − + +
WKY
SHR

Fig 7B & C

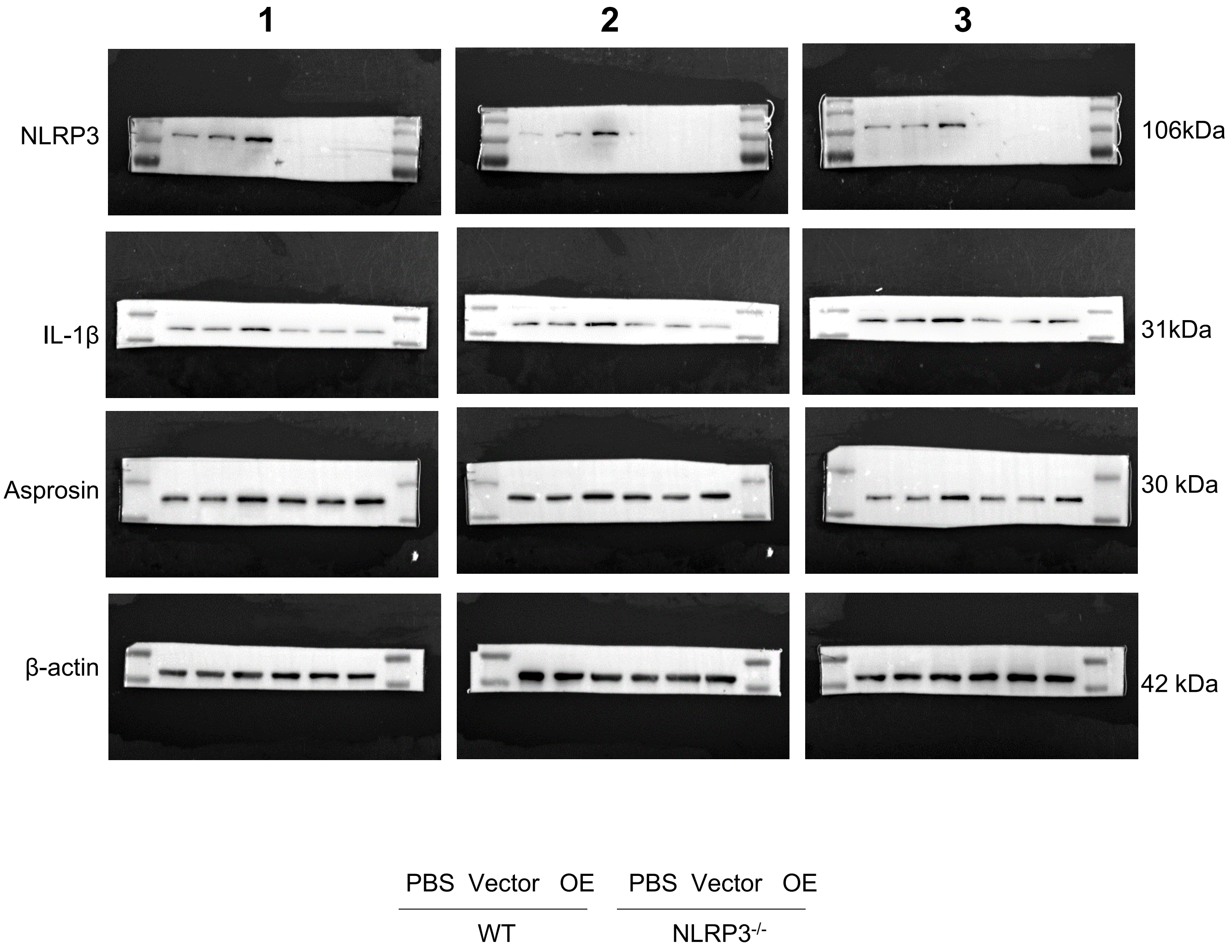

1
2
3
NLRP3
106kDa
IL-1β
31kDa
Asprosin
30 kDa
β-actin
42 kDa
PBS Vector OE
WT
PBS Vector OE
NLRP3-/-

Fig 8A

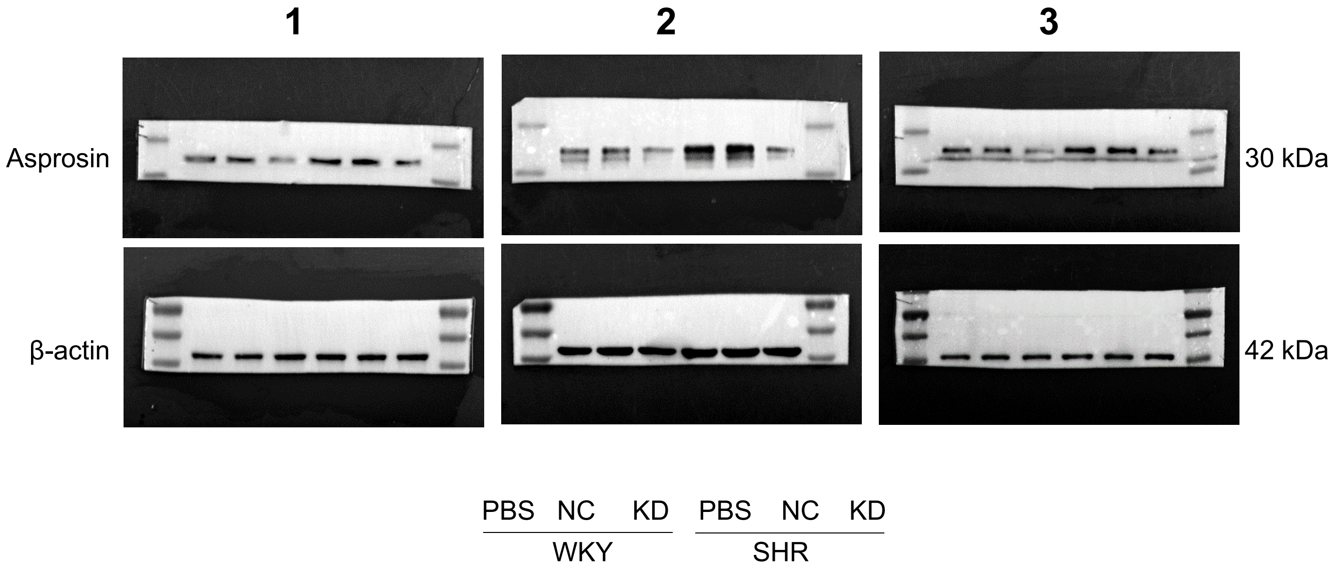

1
2
3
Asprosin
β-actin
30 kDa
42 kDa
PBS NC KD PBS NC KD
WKY
SHR

Fig 8B

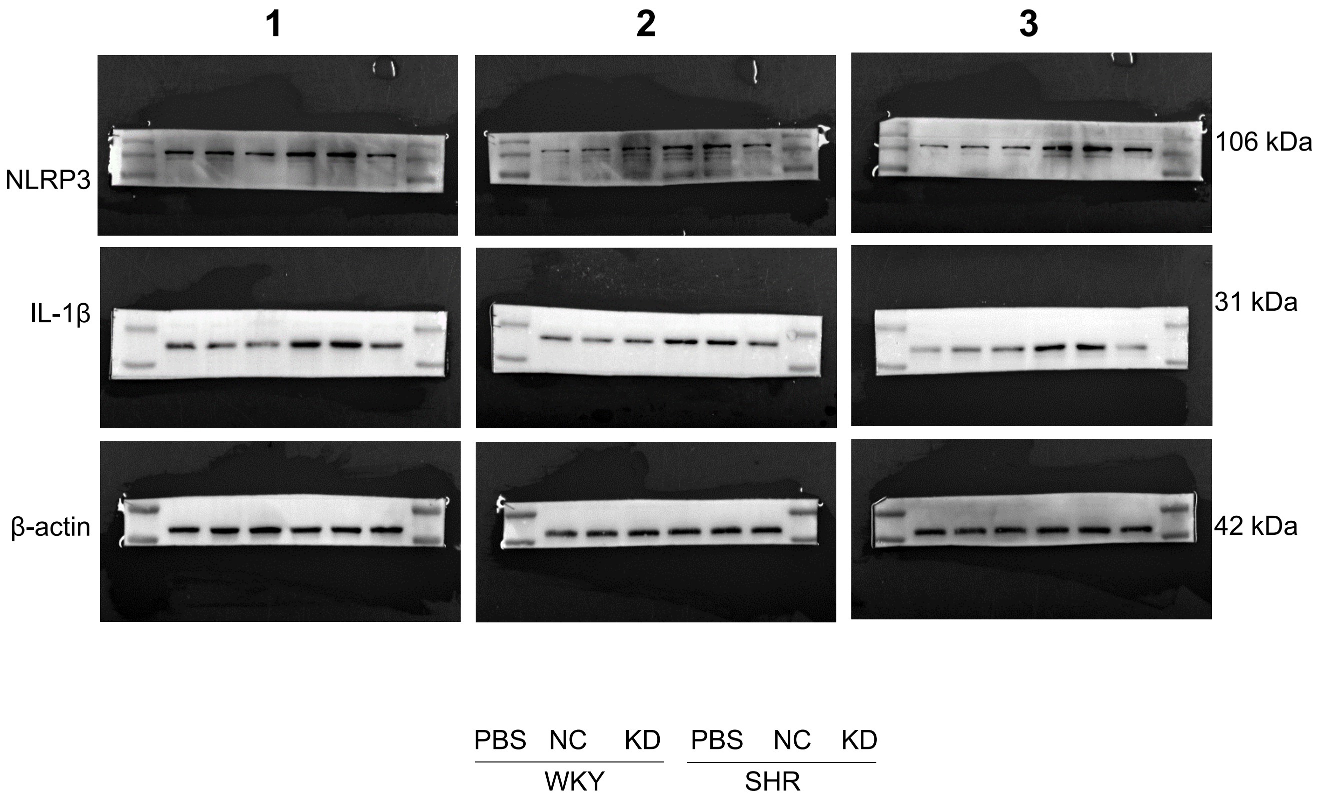

1
2
3
NLRP3
IL-1β
β-actin
106 kDa
31 kDa
42 kDa
PBS NC KD PBS NC KD
WKY
SHR
